# Combined with anti‐Nogo‐A antibody treatment, BDNF did not compensate the extra deleterious motor effect caused by large size cervical cord hemisection in adult macaques

**DOI:** 10.1111/cns.13213

**Published:** 2019-08-16

**Authors:** Marie‐Laure Beaud, Eric M. Rouiller, Jocelyne Bloch, Anis Mir, Martin E. Schwab, Eric Schmidlin

**Affiliations:** ^1^ Department of Neurosciences and Movement Sciences, Section of Medicine, Faculty of Sciences and Medicine University of Fribourg Fribourg Switzerland; ^2^ Department of Neurosurgery, Neurosurgery Clinic University Hospital of Lausanne Lausanne Switzerland; ^3^ Neuroscience Research Novartis Institute for BioMedical Research Basel Switzerland; ^4^ Brain Research Institute University of Zürich Zürich Switzerland; ^5^ Department of Biology ETH Zurich Zürich Switzerland

**Keywords:** brain‐derived neurotrophic factor, hand, manual dexterity, motor control, Nogo‐A antibody therapy, nonhuman primates, spinal cord injury

## Abstract

In spinal cord injured adult mammals, neutralizing the neurite growth inhibitor Nogo‐A with antibodies promotes axonal regeneration and functional recovery, although axonal regeneration is limited in length. Neurotrophic factors such as BDNF stimulate neurite outgrowth and protect axotomized neurons. Can the effects obtained by neutralizing Nogo‐A, inducing an environment favorable for axonal sprouting, be strengthened by adding BDNF? A unilateral incomplete hemicord lesion at C7 level interrupted the main corticospinal component in three groups of adult macaque monkeys: control monkeys (n = 6), anti‐Nogo‐A antibody‐treated monkeys (n = 7), and anti‐Nogo‐A antibody and BDNF‐treated monkeys (n = 5). The functional recovery of manual dexterity was significantly different between the 3 groups of monkeys, the lowest in the control group. Whereas the anti‐Nogo‐A antibody‐treated animals returned to manual dexterity performances close to prelesion ones, irrespective of lesion size, both the control and the anti‐Nogo‐A/BDNF animals presented a limited functional recovery. In the control group, the limited spontaneous functional recovery depended on lesion size, a dependence absent in the combined treatment group (anti‐Nogo‐A antibody and BDNF). The functional recovery in the latter group was significantly lower than in anti‐Nogo‐A antibody‐treated monkeys, although the lesion was larger in three out of the five monkeys in the combined treatment group.

## 1 INTRODUCTION

In adult mammals, following spinal cord injury (SCI), transected axons do not regenerate, resulting in persistent and often severe motor and sensory deficits. The absence of regeneration is attributed to various factors, in particular to the presence of myelin‐associated neurite growth inhibitors such as Nogo‐A in the central nervous system environment (CNS)^1^, ^2^ and/or to the presence of insufficient levels of neurotrophic factors, such as BDNF, at the site of injury. In recent years, several treatment strategies have emerged, promoting regeneration in the CNS, some with promising results. In particular, neutralizing Nogo‐A with an antibody has led to an improved functional recovery from SCI, as well as to regeneration and compensatory sprouting of corticospinal (CS) fibers in both rodents and monkeys.^1^, ^3^, ^4^, ^5^, ^6^, ^7^, ^8^, ^9^, ^10^, ^11^, ^12^ However, inhibiting Nogo‐A did not prevent the axotomized CS neurons to shrink and the extent of the CS fibers regrowth remains limited.^4^, ^7^, ^13^

Neurotrophic factors like BDNF have also been proposed as therapeutic agents to promote regeneration inside the CNS, but the effects of such treatments remain controversial. Indeed, whereas several studies concluded that BNDF delivery protects axotomized CNS neurons, promotes sprouting of injured axons, and improves functional outcome,^14^, ^15^, ^16^, ^17^, ^18^, ^19^, ^20^, ^21^, ^22^, ^23^ others reported conflicting results.^24^, ^25^, ^26^, ^27^, ^28^ In addition, in adult rats subjected to SCI a treatment combining NT‐3 and anti‐Nogo‐A antibody increased regenerative sprouting of CS fibers, whereas the combination of BDNF and anti‐Nogo‐A antibody had no effect.^28^ However, no behavioral outcome was assessed in the latter study. Moreover, the combination of BDNF and anti‐Nogo‐A antibody has not been tested yet in nonhuman primates subjected to cervical cord injury. Therefore, the aim of the present study was to investigate, in adult macaque monkeys subjected to SCI at cervical level whether the addition of BDNF can enhance the extent of functional recovery of manual dexterity prompted by anti‐Nogo‐A antibody treatment administered alone (see also Appendix S1 for rationale). We compared three groups of SCI animals: (a) monkeys treated with a control antibody, (b) monkeys treated with monoclonal anti‐Nogo‐A antibody alone, and (c) monkeys treated with BDNF combined with anti‐Nogo‐A antibody. The two first group are derived from previous studies,^6^, ^7^, ^8^, ^13^, ^29^ whereas the third group consists of 5 newly introduced monkeys as far as behavior is concerned.

Considering evidence that BDNF promotes the intrinsic capability of neuron to sprout (see above) and that anti‐Nogo‐A antibody treatment makes the CNS environment permissive for neurite outgrowth, our hypothesis is that the combined treatment (BDNF and anti‐Nogo‐A antibody) in monkeys may enhance functional recovery from SCI, as compared to single anti‐Nogo‐A antibody treatment. In our model of SCI in macaques, anti‐Nogo‐A antibody treatment administered alone yielded nearly complete functional recovery,^6^, ^8^ thus preventing to detect an improvement of functional recovery if BDNF is added to the treatment (ceiling effect). For this reason, in the present study, the strategy was to generate larger size SCI in order to produce a more extensive and extra deficit of manual dexterity. Then, if BDNF and anti‐Nogo‐A antibody combined treatments act in a synergistic manner, then one may expect a complete functional recovery of manual dexterity in spite of a large SCI.

The alternative hypothesis, considering some reported adverse effects of combining the 2 treatments (see above), is that functional recovery from SCI is reduced as compared to single anti‐Nogo‐A antibody treatment, especially if the cervical lesion is larger than in monkeys treated with anti‐Nogo‐A antibody alone.

## 2 METHODS

### 2.1 Animals origin and identification

The data are derived from experiments carried out on a total of eighteen macaque monkeys, in accordance with the Guide for Care and Use of Laboratory Animals (ISBN 0‐309‐05377‐3; 1996), and approved by local veterinary authorities, including the ethical assessment by the local (cantonal) Survey Committee on Animal Experimentation and a final acceptance delivered by the Federal Veterinary Office (BVET). The monkeys were either obtained from our own colony in our animal facility or were purchased from two certified suppliers (BioPrim, 31450 Baziège; France, and Harlan Buckshire, USA). During the course of the study, the general health condition of the monkeys was assessed by measuring their body weight regularly, generally before each daily behavioral session. During the span of the experimental period, the animals tended progressively to increase their body weight. Moreover, after SCI, the monkeys did not turn uncooperative and did not show less motivation to perform the behavioral tasks. No signs of epilepsy, aggression, or excessive fear with respect to the experimenter and animal care taker were observed.

In the present report, each monkeys’ identification code contains, for sake of clarity, a “C” or an “A” or an “AB” at fourth/fifth digit position, indicating whether the monkey was, respectively, **c**ontrol antibody‐treated, or **a**nti‐Nogo‐A antibody‐treated, or subjected to a combinatory treatment composed of **a**nti‐Nogo‐A antibody associated with the neurotrophic factor BDNF. However, during the course of the experiments, the animals had different names from which the daily experimenters could not infer in which group they belonged.

### 2.2 Behavioral assessment

At the time of the experiments reported here, all monkeys were housed in our animal facility, in rooms of 12 cubic meters, each typically containing 2‐4 monkeys free to move in the room and to interact with each other.^1^ In the morning, before behavioral testing, the animal keeper placed the monkeys in cages used to allow subsequent transfer to the primate chair (see http://www.unifr.ch/spccr). The monkeys had free access to water and were not food deprived. The rewards obtained during the behavioral tests represented the first daily access to food. After the tests, the monkeys received additional food (fruits, cereals).

**Figure 1:**
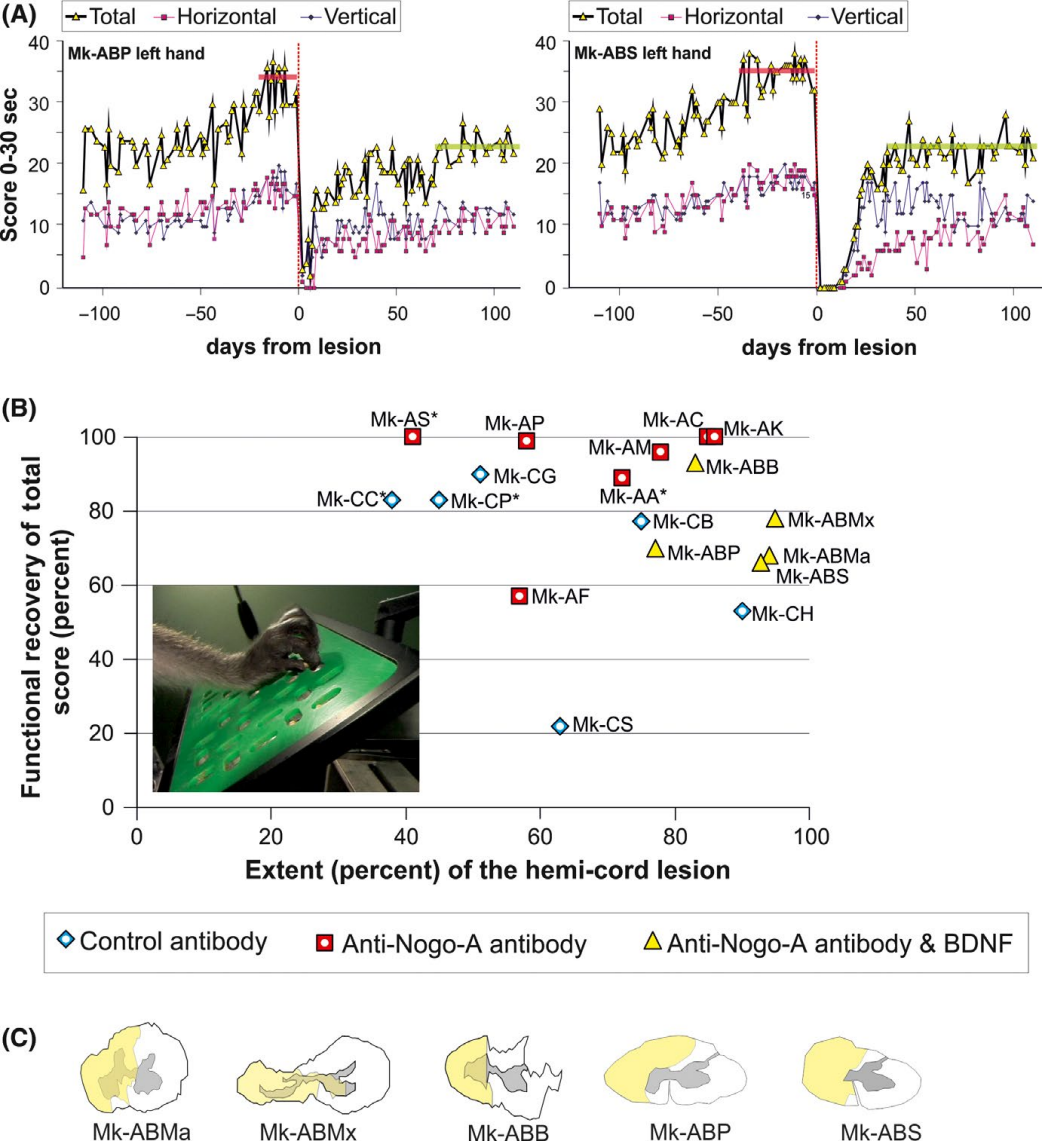
Panel A: Two examples of behavioral curves showing the retrieval scores of the hand on the side ipsilateral to the cervical cord lesion as a function of the days from lesion (before and after lesion). These data were derived from the modified Brinkman board task (number of pellets retrieved in 30 seconds), for two lesioned animals treated with anti‐Nogo‐A antibody/BDNF combination. In these graphs, day 0 corresponds to the day when the lesion was performed and is represented by a red vertical dashed line. Under each total score of each animal (yellow triangle), the scores for the vertical (blue diamonds) and horizontal (pink squares) slots were plotted separately. The two horizontal bars placed on each total score represent the prelesion plateau (red) and the postlesion plateau (yellow), respectively. Panel B: Relationship between the extent of hemicord lesion in percent and degree of functional recovery of score in % for the modified Brinkman board test for vertically and horizontally oriented slots (blue diamonds for control antibody‐treated monkeys, red squares for anti‐Nogo‐A antibody‐treated monkeys and yellow triangles for anti‐Nogo‐A antibody/BDNF‐treated monkeys). The monkeys that were already published were illustrated with an additional white circle inside their geometrical form. In this graph, one can see the tendency that the anti‐Nogo‐A antibody‐treated animals recovered better as compared to the other treated animals and independently of the size of the performed lesion. On the other hand, the animals that have received either the control antibody or the associated treatment anti‐Nogo‐A antibody/BDNF recovered less even when the lesion was smaller. A photograph, illustrating the modified Brinkman Board task used to assess manual dexterity pre‐ and postlesion, was inserted inside the graph for a better comprehension. In this image, one can see the board comprising 50 slots containing a food pellet, 25 oriented vertically and 25 horizontally. The monkey grasps the pellet in the slots by performing the precision grip (opposition of index finger and thumb). Panel C: Reconstruction of the cervical cord lesion in the 5 newly introduced monkeys subjected to the combined treatment

The dexterity of each hand was assessed separately with a finger prehension task, specifically our modified Brinkman board quantitative test.^30^, ^31^, ^32^, ^33^, ^34^ The tests were conducted using a Perspex board (10 cm x 20 cm) containing 50 randomly distributed slots, each filled with a food pellet at the beginning of the test (home designed apparatus) (Figure 1B, inset). Twenty‐five slots were oriented horizontally and twenty‐five vertically. The dimension of the slots was 15 mm long, 8 mm wide, and 6 mm deep. Retrieval of the food pellets was normally performed using the precision grip (opposition of thumb and index finger). This manual prehension dexterity task was executed daily, alternatively with one and the other hand, 4 to 5 times per week for several months before and after the unilateral cervical cord lesion. A daily behavioral session typically lasted 60 minutes. The performance of each hand was videotaped. In the present study, two parameters were assessed: (a) the retrieval score, that is, the number of wells from which the food pellets were successfully retrieved and brought to the mouth during the first 30 seconds of the test, separately for vertical wells (Figure 1A curves with blue diamonds), horizontal wells (Figure 1A curves with pink squares), or both cumulated (Figure 1A curves with yellow triangles); (b) the contact time (CT), defined as the time of contact (in seconds) between the fingers and the pellets, calculated for the first vertical well and the first horizontal well targeted by the monkey in a given daily session (as reported earlier^8^). The contact time is comparable to the prehension time as introduced by Nishimura et al^35^ for a different grasping task. The CT reflects the true capacity of performing the precision grip using the index finger and the thumb; it is defined as the time separating the first contact of the index finger with the food pellet and the final successful grasp of the pellet utilizing pad‐to‐pad opposition. As compared to the score, the CT is restricted to manual dexterity per se as it does not include the time to transport the pellet to the mouth and the time to bring back the arm to the board to catch the next pellet.

After a period ranging from 1 to 5 months across individuals, corresponding to a phase of initial training,^36^ the monkeys reached and maintained a stable level of performance (prelesion plateau). Once reaching this level, they were subjected to the unilateral cervical cord lesion. After lesion and in absence of treatment (n = 6 control antibody monkeys), there was a dramatic loss of manual dexterity, as reflected by a score of zero, followed by a progressive functional recovery up to a variable extent across individuals.^6^, ^8^, ^32^, ^33^ The duration of the functional recovery period ranged from 28 to 76 days across individuals (n = 6 control antibody monkeys), after which a stable level of recovered performance was reached, corresponding to a postlesion plateau (see Ref.^37^ for the quantitative criterion defining the plateau onset). In the anti‐Nogo‐A antibody group, the duration of functional recovery ranged from 19 to 43 days whereas in the combined treatment group the range was 20 to 95 days.

**Figure 2:**
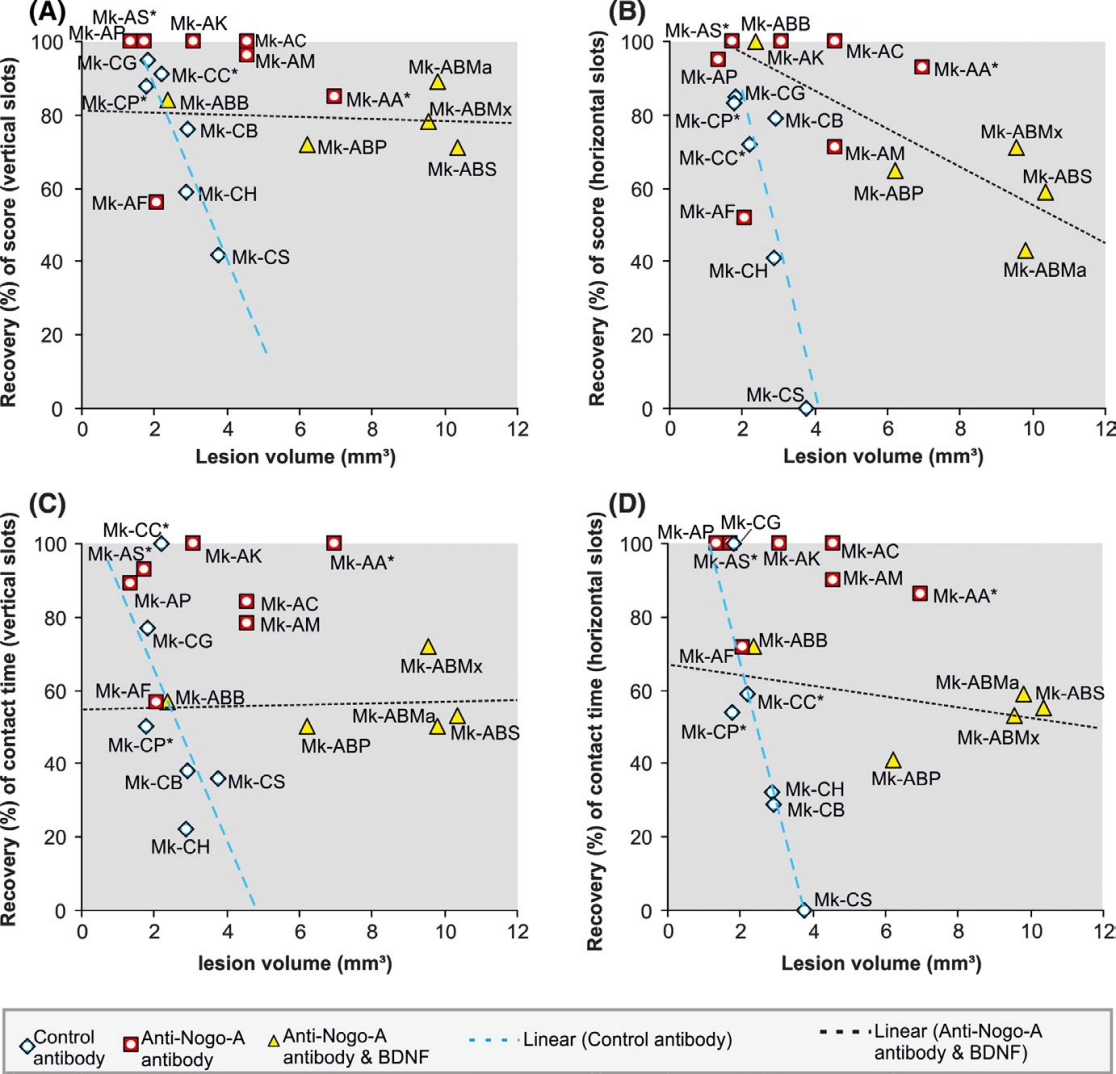
Relationship between the behavioral parameters score and contact time as function of the estimated volume of the cervical cord lesion in mm^3^. Panels A and B: Relationship between the degree of functional recovery of score (as a percentage) for the modified Brinkman board test and the estimated volume of the cervical lesion for (A) vertically and (B) horizontally oriented slots (blue diamonds for control antibody‐treated monkeys, red squares for anti‐Nogo‐A antibody‐treated monkeys and yellow triangles for anti‐Nogo‐A antibody/BDNF‐treated monkeys). Panels C and D: Relationship between the degree of functional recovery of contact time needed for the first successful retrieval and the estimated volume of the cervical lesion, for (C) vertically and (D) horizontally oriented slots (blue diamonds for control antibody‐treated monkeys, red squares for anti‐Nogo‐A antibody‐treated monkeys and yellow triangles for anti‐Nogo‐A antibody/BDNF‐treated monkeys). In each panel, the dotted blue line and the black dotted line represent the linear regression line calculated for the score or contact time and the estimated volume of the lesion for the group of control antibody‐treated monkeys (blue diamonds) and anti‐Nogo‐A antibody/BDNF‐treated monkeys (yellow triangles), respectively. In four monkeys (*), the dorsolateral funiculus was not completely transected and the monkey Mk‐AK differ from the others because postlesion treatment was delayed by 1 week. See Tables 2 and 3 for statistical comparisons between the group of monkeys treated with anti‐Nogo‐A antibody and BDNF versus the other 2 groups of monkeys

The degree of functional recovery was established by calculating the ratio in percent of the average retrieval score at postlesion plateau to that observed at prelesion plateau. In addition to the average score value, the CT of the first vertical and first horizontal slots targeted by monkeys was measured in each daily session at prelesion and postlesion plateaux. In order to minimize the impact of outliers, the prelesion and postlesion CTs were represented by their median value (Figure 2C,D). Considering that a good performance is reflected by a short CT, the degree of functional recovery derived from the CTs was expressed quantitatively as the ratio in percent of the prelesion median CT to the postlesion median CT. For measures of both degrees of functional recovery, derived from the score or the CT, respectively, if the calculated values exceeded 100% (ie, postlesion performance was better than prelesion performance), the recovery was considered to be complete and therefore expressed quantitatively as 100% (though this was rare). The percentage of functional recovery was the main parameter used to assess the effect of a treatment after SCI (anti‐Nogo‐A antibody alone or combined anti‐Nogo‐A antibody‐BDNF) in comparison with the control antibody‐treated monkeys (Figures 1 and 2).

The overall duration of the experiment was quite variable across monkeys, especially the pretraining phase^36^ and the prelesion phase as well. However, considering the relevant portion of the prelesion phase with stable behavior, the duration of recovery, and a well‐stabilized postlesion plateau of a few months at least, the overall duration of the experiment ranged between about 200 and 350 days.

### 2.3 Surgical procedures

All surgical procedures are the same as previously described^6^, ^7^, ^8^, ^29^, ^38^ and can be found in the Appendix S1. It includes anesthesia, postsurgery care of the animals, procedure to perform the cervical cord lesion at C7.

### 2.4 Treatments and groups of monkeys

At the end of the surgery during which the cervical cord lesion was performed, the tip of one or two catheters were inserted intrathecally in the vicinity of the lesion site. The other side of the catheters was attached to an osmotic pump which could deliver a volume of 2 mL of control antibody (control group n = 6) or of anti‐Nogo‐A antibody (anti‐Nogo‐A group n = 7) or of BDNF and anti‐Nogo‐A antibody (combined treatment group n = 5). The 5 monkeys of the combined treatment group were implanted with two pumps, one delivering BDNF (1.4 mg in 4 weeks) and the second pump the anti‐Nogo‐A antibody (14.8 mg in 4 weeks). Further detail on the treatment procedures and additional monkeys not included in the present analysis can be found in the Appendix S1.

### 2.5 Histology

The histological procedures are described in the Appendix S1, in particular the method to assess the extent and volume of the spinal cord lesion.

### 2.6 Statistical analysis

The statistical analysis aimed at comparing the various variables reflecting the extent of functional recovery (score or CT) across the three groups of monkeys as a function of the volume of lesion (Tables 2 and 3). For the score data, the variables tested were the score obtained in the vertical slots (VSc) and the score obtained in the horizontal slots (HSc). For the CT, the variables were the recovery of the median CT in the vertical slots (VCT) and the median CT in the horizontal slots (HCT). The statistical analysis was an extension of that performed earlier,^8^ based on the original multivariate nonparametric approach proposed by Oja and Randles.^39^ The statistical test was extended for a use with R^40^ and applied to the present behavioral data with the contribution of the Mathematics Department of University of Fribourg.

A first trivariate comparison was performed between the 3 groups of monkeys, with the hypothesis H_o_ that there is no difference between them (Table 2). Second, a bivariate analysis was conducted to compare one by one the 3 groups of monkeys, with the same hypothesis H_o_ on all combinations of two groups, comparing the newly introduced group anti‐Nogo‐A antibody and BDNF versus each of the other two groups (Table 3). For the latter one‐by‐one group comparison, when the functional recovery was not dependent on the lesion volume (not statistically significant correlation), a univariate nonparametric test was applied (Mann‐Whitney rank‐sum test; see Table 3). The trivariate analysis was also applied to the data displayed in Figure 1, in order to compare the functional recovery of total score across the 3 groups of monkeys as a function of lesion extent expressed in % (see results below).

## 3 RESULTS

### 3.1 Cervical cord lesion

The location and extent of the lesion were assessed in each monkey by reconstructing the lesion site from histological sections. The cervical lesions in the control antibody monkeys (n = 6) and the anti‐Nogo‐A antibody monkeys (n = 7) were all illustrated in a previous report^8their Fig. 1^. From these data, the extent of their lesion expressed either in percentage of hemisection or in volume (mm^3^) was derived (see Table 1 in Ref.^8^) and used in the present study (Figures 1 and 2). The cervical lesions in the newly introduced group of monkeys (n = 5) subjected to the combined treatment (anti‐Nogo‐A antibody and BDNF) are illustrated in Figure 1C, with their extent listed in Table 1. The aim to produce larger cervical lesion in these 5 combined treatment monkeys was largely achieved as three of these animals (Mk‐ABMa; Mk‐ABMx, Mk‐ABS) had a lesion bigger than all monkeys in the two other groups. The lesion size in the last two combined treatment monkeys (Mk‐ABB and Mk‐ABP) overlapped with the other groups (Figures 1B and 2), although Mk‐ABP still stands among the largest lesions when expressed in mm^3^. The average lesion sizes expressed in % of hemisection and in volume were as follows. In the control antibody group, it was 60.3% and 2.556 mm^3^; in the anti‐Nogo‐A antibody group 68.3% and 3.479 mm^3^; and in the anti‐Nogo‐A and BDNF group 88.4% and 7.658 mm^3^.

**Table 1:** List of the new monkeys included in the present study with respect to behavior, with identification code

ID code	Mk‐ABMx	Mk‐ABMa	Mk‐ABB	Mk‐ABS	Mk‐ABP
Species	fasc.	fasc.	fasc.	fasc.	fasc.
Sex	Male	Male	Male	Male	Male
Treatment	BNDF & hNogo	BNDF & hNogo	BNDF & hNogo	BNDF & hNogo	BNDF & hNogo
Weight	5.4	3.5	5.3	4.2	4.6
“Experimenter Blind” procedure	Yes	Yes	Yes	Yes	Yes
Hemisection extent (%)	95	94	83	93	77
Volume of lesion (mm^3^)	9.55	9.81	2.36	10.37	6.2
Functional recovery (%)					
Score (vert)	78	89	84	71	72
Score (horiz)	71	43	100	59	65
Contact time (vert)	72	50	57	53	50
Contact time (horiz)	53	59	72	55	41
Completeness of dlf section	Yes	Yes	Yes	Yes	Yes
Stock farming	BioPrim	BioPrim	BioPrim	Buckshire USA	Buckshire USA

Note

At the time of the experiment, monkeys were assigned names that did not allow experimenters to determine whether the animal was infused with the control (n = 2 excluded from the analysis) or the anti‐Nogo‐A antibody (n = 2 not considered here) or the anti‐Nogo‐A antibody combined with BDNF. New names (codes) were assigned to the monkeys during the writing of the manuscript to improve its readability.

Functional recovery (expressed in % for the behavioral parameter “score”) was assessed here based on the modified Brinkman board task, by comparing the performance pre‐ and postlesion, as explained in detail in the Materials and Methods section.

Abbreviations: dlf, dorsolateral funiculus; fasc., Macaca fascicularis; hNogo, a monoclonal anti‐Nogo‐A antibody which was raised by immunization with the whole Nogo‐A‐specific region of the human Nogo‐A sequence; horiz, horizontal; vert, vertical.

### 3.2 Postlesion recovery of manual dexterity: total score versus hemisection extent in %

The manual dexterity was evaluated pre‐ and postlesion using the modified Brinkman board task. The manual dexterity score varied in a characteristic way during the time course of the experiment (Figure 1A), exhibiting first a learning phase with improving score, preceding a prelesion plateau corresponding to a stable reference level of manual dexterity performance (Figure 1A; red lines). Following this phase of stable performance, a unilateral cervical cord lesion affected the hand with which the monkey reached the highest score (dominant hand: see Ref.^34^). After the lesion, the score fell down drastically reaching zero during several days, before a progressive reincrease, corresponding to the functional recovery phase (Figure 1A). Finally, the score stabilized again into a postlesion plateau, reflecting the final extent of functional recovery of manual dexterity (Figure 1A; yellow lines).

To assess how functional recovery is related to the size of the lesion, the extent of functional recovery (ratio of postlesion versus prelesion total score plateaux in percent) was plotted as a function of the extent of the hemisection expressed in percent of the hemicord injured (Figure 1B; see also Table 1 and Ref.^8^ for individual values). As expected and confirming previous data,^6^, ^8^ control antibody‐treated monkeys exhibited a spontaneous functional recovery of total score somewhat dependent on the lesion extent, with lower recovery values for lesion extents larger than 60% than for smaller lesions (<60%; blue diamonds in Figure 1B). In the anti‐Nogo‐A antibody‐treated monkeys (red squares in Figure 1B), the functional recovery of total score was more prominent than in control monkeys and largely independent of the lesion extent (see however Figure 2 for correlation statistical analyses). A trivariate nonparametric statistical analysis on the data displayed in Figure 1B, as described in the methods above, yielded a *P* value of .022, indicating that the three groups of monkeys are significant distinct, with respect to the functional recovery of total score versus cervical cord lesion extent.

In the newly introduced group of combined treatment monkeys (n = 5, yellow triangles in Figure 1B), there was no apparent relationship between the functional recovery of total score and the lesion extent (see, however, Figure 2 for correlation statistical analyses). A comparison of the 2 groups (anti‐Nogo‐A antibody versus combined treatment: red squares vs yellow triangles in Figure 1B) showed a nonstatistically significant difference of functional recovery of total score (Mann‐Whitney rank‐sum test *P* = .073; see, however, separate data for vertical and horizontal slots below in Figure 2 and Tables 2 and 3).

**Table 2:** Trivariate nonparametric statistical comparison (see Freund et al^8^; Oja and Randall^39^; Oja^40^) between the three groups of monkeys: (a) control antibody; (b) anti‐Nogo‐A antibody; (c) anti‐Nogo‐A antibody and BDNF for the lesion volume and every other variables (score vertical slots = VSc; score horizontal slots = HSc; median CT vertical slots = VCT; median CT horizontal slots = HCT), corresponding to the four panels of Figure 2

Volume of lesion (mm^3^)	
Condition	*P*‐value
Recovery of score vertical slots (Figure 2A)	.013
Recovery of score horizontal slots (Figure 2B)	.007
Recovery of CT vertical slots (Figure 2C)	.003
Recovery of CT horizontal slots (Figure 2D)	.001

**Table 3:** One‐by‐one statistical comparisons across the same three groups taken two by two (all combinations of group 3 versus group 1 or group 3 versus group 2). In case the functional recovery in the two groups compared were independent from the volume of lesion, the statistical test was univariate (Mann‐Whitney rank‐sum test), whereas it was a bivariate nonparametric statistical test (Freund et al^8^; Oja^40^) when one of the group exhibited a dependence of the functional recovery with respect to the lesion volume

*P* values		
	Control antibody	Anti‐Nogo‐A antibody
Anti‐Nogo‐A + BDNF VSc^2A^	0.032†	0.073*
Anti‐Nogo‐A + BDNF HSc^2B^	0.019†	0.073†
Anti‐Nogo‐A + BDNF VCT^2C^	0.037†	0.005*
Anti‐Nogo‐A + BDNF HCT^2D^	0.028†	0.003*

Note

See Freund et al^8^ for comparisons between the control antibody and the anti‐Nogo‐A antibody monkey groups (their Table 2), not repeated here. The here newly introduced group anti‐Nogo‐A antibody and BDNF‐treated monkeys, listed in the leftmost column is compared for the same functional recovery parameters (VSc vs VSc, HSc vs HSc, VCT vs VCT, HCT vs HCT) with the other two groups of monkeys (middle and rightmost columns).

^2A, 2B, 2C, 2D^Refers to the panels A,B,C, and D of Figure 2.

* Univariate comparison (Mann‐Whitney rank‐sum test).

^†^ Bivariate statistical comparison, as described in Freund et al^8^ and Oja.^40^

### 3.3 Postlesion recovery of manual dexterity: separate scores and CTs for Vertical and Horizontal slots versus cervical cord lesion volume in mm^3^

The movement strategy for picking food pellets out of the horizontal and vertical slots differs,^8^, ^33^ the positioning of the fingers inside the horizontal slots requiring a rotation of the wrist, not needed for the vertical slots. The impact of a lesion on the capacity to empty horizontal slots reflects this difference and is often more pronounced than that observed for the vertical slots. The behavioral scores for the vertical and for the horizontal slots were established separately (see blue diamonds and red squares in Figure 1A, respectively). As a consequence, the functional recovery in % was computed separately for the vertical and horizontal slots (Figure 2).

The measure of the extent of the lesion in percent on a single histological section taken at the middle of the lesion only (Figure 1C) gives information about the degree of the interruption of pathways running along the spinal cord. To also take into account the overall magnitude of tissue damage, the functional recovery of scores was plotted as a function of the estimated volume of the lesion in mm^3^, for both slot orientations (Figure 2A,B). Similarly, functional recovery of contact time was plotted as a function of the estimated volume of the lesion in Figure 2C,D, for the vertical and horizontal slots, respectively.

A trivariate nonparametric statistical analysis was performed, as explained in the methods, on the data displayed in each of the four panels in Figure 2. The results of this trivariate analysis showed that, for each of the four functional recovery parameters as a function of lesion volume, the three groups of monkeys were significantly distinct from each other (Table 2). In other words, the functional recovery of score or CT was significantly different when a control antibody was applied, as compared to administration of the anti‐Nogo‐A antibody alone or to administration of the combined treatment (anti‐Nogo‐A antibody and BDNF).

As previously reported^8their Fig. 5^ and as expected, the four panels in Figure 2(A‐D) show that the functional recovery in % reflected by four distinct parameters are indeed inversely correlated with lesion volume in the control monkeys (blue diamonds and blue dashed regression lines in Figure 2). In contrast, the functional recoveries in the anti‐Nogo‐A antibody‐treated monkeys were not correlated to the lesion volume (Figure 2 red squares; see Ref.^8^ for negative correlation statistical data). As far as the combined treatment group is concerned (yellow triangles and black dashed regression lines in Figure 2), the functional recovery of vertical score, vertical CT, and horizontal CT was not correlated to the lesion volume (*R* = −0.176, *R* = 0.102, *R* = −0.428, respectively; all three with a *P* value > .05). Only the recovery of the horizontal score was related to the lesion volume in the combined treatment monkeys (Figure 2B, yellow triangles; *R *= −0.84, *P* < .05).

Previous data demonstrated that the anti‐Nogo‐A antibody enhanced functional recovery from cervical cord lesion,^8^ representing a strong incentive to initiate clinical trials.^41^ In other words, the functional recovery from cervical cord hemisection was significantly better when the anti‐Nogo‐A antibody was delivered as compared to a control antibody.^6^, ^8^ The main aim of the present study was to compare the anti‐Nogo‐A antibody‐treated monkeys of Freund et al^8^ with the newly introduced combined treatment monkeys (anti‐Nogo‐A antibody and BDNF). In panels A, C, and D of Figure 2, as the data points (red squares and yellow triangles) were independent of the lesion volume, a univariate nonparametric comparison of functional recovery was performed (Table 3, p‐values with an asterisk). For both vertical CT and horizontal CT (panels C and D in Figure 2: red squares versus yellow triangles), there was a statistically significant difference of functional recovery between the 2 groups: *P* = .005 and *P* = .003, respectively. It was only a trend when considering the recovery of the vertical score (Figure 2 panel A: red squares vs yellow triangles): *P* = .073. For the data in panel B of Figure 2, as the functional recovery in the combined treatment group was correlated to the lesion volume, a bivariate nonparametric analysis was conducted, showing also a trend: *P* = .073). From these two by two comparisons, it can be concluded that, based on the contact time (CT) data, the functional recovery was significantly lower in the combined treatment group as compared to the anti‐Nogo‐A antibody‐treated group (Table 3). For the functional recovery of score, it was also lower in the combined group of monkeys as compared to the anti‐Nogo‐A antibody‐treated group, but this was only a trend.

Finally, a statistical comparison was made between the newly introduced group of monkeys subjected to the combined treatment and the control group of monkeys, for the data displayed in the four panels of Figure 2. The p‐values listed in Table 3 (middle column), yielded from two by two bivariate nonparametric tests, were all statistically significant (*P* < .05). It can be concluded that the four parameters of functional recovery were better in the combined treatment group as compared to the control group.

## 4 CONCLUSION

The main result of the present study is that the 3 groups of monkeys subjected to cervical cord hemisection and the administration of 3 different treatments significantly differ with respect to their four functional recovery parameters plotted as a function of lesion volumes (Figure 2 and Table 2). The present report is an extension of a previous study,^8^ which demonstrated that an intrathecal treatment with the anti‐Nogo‐A antibody led to enhanced functional recovery of manual dexterity in adult macaques, as compared to control antibody‐treated monkeys, following hemisection of the cervical spinal cord. We report here on a newly introduced third group of monkeys, treated with a combination of anti‐Nogo‐A antibody and BDNF, also after cervical cord hemisection, although somewhat bigger than in the previous two groups of monkeys (Figure 2). As demonstrated by 3 monkeys with the combined treatment and subjected to a large lesion, the addition of BDNF did not compensate in term of functional recovery for the excess in lesion volume. These data in monkeys Mk‐ABMx, Mk‐ABMa, and Mk‐ABS rather suggest that the functional recovery was most likely reduced by the presence of BDNF, as compared to a single treatment limited to anti‐Nogo‐A antibody alone (Figure 2). Such tentative conclusion is supported by the monkeys Mk‐ABP and Mk‐ABB, overlapping the group of anti‐Nogo‐A antibody‐treated monkeys with respect to lesion volume, showing also a diminished functional recovery as compared to the latter group (Figure 2). Due to the small sample (two overlapping monkeys), the conclusion that BDNF does not provide strong synergistic effect when added to the anti‐Nogo‐A antibody in order to promote functional recovery after cervical cord lesion remains only tentative at that step and would need to be confirmed on a larger number of monkeys. On the quantitative point of view nevertheless, the counteraction of BDNF does not totally suppress the beneficial effect of the anti‐Nogo‐A antibody, as the combined treatment group of monkeys still recovered better than the control antibody‐treated monkeys (Figure 2; Table 3). Note that one monkey of the combined treatment group (Mk‐ABB) exhibited a rather good functional recovery, higher than the other four monkeys of the same group (Figure 2). Although the limited volume of lesion in Mk‐ABB may explain this observation (2.4 mm^3^ versus > 6 mm^3^ in the other 4 monkeys), there is also the suspicion that the lesion may be a bit more caudal than the other monkeys. Its scores did not drop all the way to zero immediately postlesion and the functional recovery was clearly faster (14 days) than in the other animals (20 days in Mk‐ABP; 26 days in Mk‐ABS; 47 days in Mk‐ABMa; 95 days in Mk‐ABMx).

In conclusion, our findings suggest that adding BDNF did not potentiate the effects leading to enhanced functional recovery acquired when the anti‐Nogo‐A antibody was given alone. It is unlikely that the antibodies and BDNF interacted directly. Rather, they may exert independent effects on different parts of the nervous system. BDNF is known to strongly act on dorsal root ganglia and their fibers, pain, or spasticity (even below detection limit) could be at the origin of decreased hand function. More generalized CNS effects are also possible. Thus, the rationale to use a more broadly acting neurotrophic factor (BDNF rather than NT‐3) did not yield the expected benefit.

## CONFLICT OF INTEREST

The anti‐Nogo‐A antibody was provided by Novartis AG (Basel, Switzerland).

## Data Availability

Access to the behavioral raw data (video sequences), as well as to histological data (slides), can be provided upon request to the corresponding author.

## ENDNOTE

1 A new Swiss regulation has been introduced in September 2010 requesting now a volume of 45 cubic meters at least to be given to a group of 2 to 5 adult macaque monkeys.

## References

[1] Schwab ME . Nogo and axon regeneration. Curr Opin Neurobiol. 2004;14:118‐124. 15018947 10.1016/j.conb.2004.01.004

[2] Schwab ME . Functions of Nogo proteins and their receptors in the nervous system. Nat Rev Neurosci. 2010;11:799‐811. 21045861 10.1038/nrn2936

[3] Bregman BS , Kunkel‐Bagden E , Schnell L , Dai HN , Gao D , Schwab ME . Recovery from spinal cord injury mediated by antibodies to neurite growth inhibitors. Nature. 1995;378:498‐501. 7477407 10.1038/378498a0

[4] Brösamle C , Huber AB , Fiedler M , Skerra A , Schwab ME . Regeneration of lesioned corticospinal tract fibers in the adult rat induced by a recombinant, humanized IN‐1 antibody fragment. J Neurosci. 2000;20:8061‐8068. 11050127 10.1523/JNEUROSCI.20-21-08061.2000 PMC6772740

[5] Fouad K , Klusman I , Schwab ME . Regenerating corticospinal fibers in the Marmoset (*Callitrix jacchus*) after spinal cord lesion and treatment with the anti‐Nogo‐A antibody IN‐1. Eur J Neurosci. 2004;20:2479‐2482. 15525289 10.1111/j.1460-9568.2004.03716.x

[6] Freund P , Schmidlin E , Wannier T , et al. Rouiller EM Nogo‐A‐specific antibody treatment enhances sprouting and functional recovery after cervical lesion in adult primates. Nature Med. 2006;12:790‐792. 16819551 10.1038/nm1436

[7] Freund P , Wannier T , Schmidlin E , et al. Anti‐Nogo‐A antibody treatment enhances sprouting of corticospinal axons rostral to a unilateral cervical spinal cord lesion in adult macaque monkey. J Comp Neurol. 2007;502:644‐659. 17394135 10.1002/cne.21321

[8] Freund P , Schmidlin E , Wannier T , et al. Anti‐Nogo‐A antibody treatment promotes recovery of manual dexterity after unilateral cervical lesion in adult primates–re‐examination and extension of behavioral data. Eur J Neurosci. 2009;29:983‐996. 19291225 10.1111/j.1460-9568.2009.06642.x PMC2695186

[9] Gonzenbach RR , Schwab ME . Disinhibition of neurite growth to repair the injured adult CNS: focusing on Nogo. Cell Mol Life Sci. 2008;65:161‐176. 17975707 10.1007/s00018-007-7170-3 PMC11131900

[10] Liebscher T , Schnell L , Schnell D , et al. Nogo‐A antibody improves regeneration and locomotion of spinal cord‐injured rats. Ann Neurol. 2005;58:706‐719. 16173073 10.1002/ana.20627

[11] Schnell L , Schwab ME . Axonal regeneration in the rat spinal cord produced by an antibody against myelin‐associated neurite growth inhibitors. Nature. 1990;343:269‐272. 2300171 10.1038/343269a0

[12] Thallmair M , Metz G , Z'Graggen WJ , et al. Neurite growth inhibitors restrict plasticity and functional recovery following corticospinal tract lesions. Nature Neurosci. 1998;1:124‐131. 10195127 10.1038/373

[13] Beaud ML , Schmidlin E , Wannier T , et al. Anti‐Nogo‐A antibody treatment does not prevent cell body shrinkage in the motor cortex in adult monkeys subjected to unilateral cervical cord lesion. BMC Neurosci. 2008;9:5. 18194520 10.1186/1471-2202-9-5 PMC2242790

[14] Bregman BS , McAtee M , Dai HN , Kuhn PL . Neurotrophic factors increase axonal growth after spinal cord injury and transplantation in the adult rat. Exp Neurol. 1997;148:475‐494. 9417827 10.1006/exnr.1997.6705

[15] Brock JH , Rosenzweig ES , Blesch A , et al. Local and remote growth factor effects after primate spinal cord injury. J Neurosci. 2010;30:9728‐9737. 20660255 10.1523/JNEUROSCI.1924-10.2010 PMC2927098

[16] Giehl KM , Tetzlaff W . BDNF and NT‐3, but not NGF, prevent axotomy‐induced death of rat corticospinal neurons *in vivo* . Eur J Neurosci. 1996;8:1167‐1175. 8752586 10.1111/j.1460-9568.1996.tb01284.x

[17] Giehl KM , Schütte A , Mestres P , Yan QA . The survival‐promoting effect of glial cell line‐derived neurotrophic factor on axotomized corticospinal neurons *in vivo* is mediated by an endogenous brain‐derived neurotrophic factor mechanism. J Neurosci. 1998;18:7351‐7360. 9736655 10.1523/JNEUROSCI.18-18-07351.1998 PMC6793255

[18] Hammond E , Tetzlaff W , Mestres P , Giehl KM . BDNF, but not NT‐3, promotes long‐term survival of axotomized adult rat corticospinal neurons *in vivo* . NeuroReport. 1999;10:2671‐2675. 10574390 10.1097/00001756-199908200-00043

[19] Hiebert GW , Khodarahmi K , McGraw J , Steeves JD , Tetzlaff W . Brain‐derived neurotrophic factor applied to the motor cortex promotes sprouting of corticospinal fibers but not regeneration into a peripheral nerve transplant. J Neurosci Res. 2002;69:160‐168. 12111797 10.1002/jnr.10275

[20] Kamei N , Tanaka N , Oishi Y , et al. NGF released from transplanted neural progenitor cells promote corticospinal axon growth in organotypic cocultures. Spine. 2007;32(12):1272‐1278. 17515814 10.1097/BRS.0b013e318059afab

[21] Namiki J , Kojima A , Tator CH . Effect of brain‐derived neurotrophic factor, nerve growth factor, and neurotrophin‐3 on functional recovery and regeneration after spinal cord injury in adult rats. J Neurotrauma. 2000;17:1219‐1230. 11186234 10.1089/neu.2000.17.1219

[22] Vavrek R , Girgis J , Tetzlaff W , Hiebert GW , Fouad K . BDNF promotes connections of corticospinal neurons onto spared descending interneurons in spinal cord injured rats. Brain. 2006;129:1534‐1545. 16632552 10.1093/brain/awl087

[23] Zhou L , Shine HD . Neurotrophic factors expressed in both cortex and spinal cord induce axonal plasticity after spinal cord injury. J Neurosci Res. 2003;74:221‐226. 14515351 10.1002/jnr.10718

[24] Iarikov DE , Kim BG , Dai HN , McAtee M , Kuhn PL , Bregman BS . Delayed transplantation with exogenous neurotrophin administration enhances plasticity of corticofugal projections after spinal cord injury. J Neurotrauma. 2007;24:690‐702. 17439351 10.1089/neu.2006.0172

[25] Lu P , Blesch A , Tuszynski MH . Neurotrophism without neurotropism: BDNF promotes survival but not growth of lesioned corticospinal neurons. J Comp Neurol. 2001;436:456‐470. 11447589 10.1002/cne.1080

[26] Nakahara Y , Gage FH , Tuszynski MH . Grafts of fibroblasts genetically modified to secrete NGF, BDNF, NT‐3, or basic FGF elicit differential responses in the adult spinal cord. Cell Transplant. 1996;5:191‐204. 8689031 10.1177/096368979600500209

[27] Schnell L , Schwab ME . Sprouting and regeneration of lesioned corticospinal tract fibres in the adult rat spinal cord. Eur J Neurosci. 1993;5:1156‐1171. 8281320 10.1111/j.1460-9568.1993.tb00970.x

[28] Schnell L , Schneider R , Kolbeck R , Barde YA , Schwab ME . Neurotrophin‐3 enhances sprouting of corticospinal tract during development and after adult spinal cord lesion. Nature. 1994;367:170‐173. 8114912 10.1038/367170a0

[29] Beaud ML , Rouiller EM , Bloch J , et al. Invasion of lesion territory by regenerating fibers after spinal cord injury in adult macaque monkeys. Neurosci. 2012;227:271‐282. 10.1016/j.neuroscience.2012.09.052 23036616

[30] Rouiller EM , Yu XH , Moret V , Tempini A , Wiesendanger M , Liang F . Dexterity in adult monkeys following early lesion of the motor cortical hand area: the role of cortex adjacent to the lesion. Eur J Neurosci. 1998;10:729‐740. 9749734 10.1046/j.1460-9568.1998.00075.x

[31] Liu Y , Rouiller EM . Mechanisms of recovery of dexterity following unilateral lesion of the sensorimotor cortex in adult monkeys. Exp Brain Res. 1999;128:149‐159. 10473753 10.1007/s002210050830

[32] Schmidlin E , Wannier T , Bloch J , Rouiller EM . Progressive plastic changes in the hand representation of the primary motor cortex parallel incomplete recovery from a unilateral section of the corticospinal tract at cervical level in monkeys. Brain Res. 2004;1017:172‐183. 15261113 10.1016/j.brainres.2004.05.036

[33] Schmidlin E , Kaeser M , Gindrat A‐D et al. Behavioral assessment of manual dexterity in non‐human primates. J Vis Exp. 2011;57:pii:3258. 10.3791/3258 PMC3308590 22105161

[34] Chatagny P , Badoud S , Kaeser M , et al. Distinction between hand dominance and hand preference in primates: a behavioral investigation of manual dexterity in nonhuman primates (macaques) and human subjects. Brain Behav. 2013;3:575‐595. 24392278 10.1002/brb3.160 PMC3869985

[35] Nishimura Y , Onoe H , Morichika Y , Perfiliev S , Tsukada H , Isa T . Time‐dependent central compensatory mechanisms of finger dexterity after spinal cord injury. Science. 2007;318:1150‐1155. 18006750 10.1126/science.1147243

[36] Kaeser M , Chatagny P , Gindrat AD , et al. Variability of manual dexterity performance in non‐human primates (*Macaca fascicularis*). Int J Comp Psychol. 2014;27:295‐325.

[37] Kaeser M , Brunet JF , Wyss A , et al. Autologous adult cortical cell transplantation enhances functional recovery following unilateral lesion of motor cortex in primates: a pilot study. Neurosurgery. 2011;68(5):1405‐1417. discussion 1416–1417. 21273922 10.1227/NEU.0b013e31820c02c0

[38] Wannier T , Schmidlin E , Bloch J , Rouiller EM . A unilateral section of the corticospinal tract at cervical level in primate does not lead to measurable cell loss in motor cortex. J Neurotrauma. 2005;22:703‐717. 15941378 10.1089/neu.2005.22.703

[39] Oja H , Randles RH . Multivariate nonparametric tests. Stat Sci. 2004;19:598‐605.

[40] Oja H. Multivariate nonparametric methods with R: an approach based on spatial signs and ranks, 1st Edn New York, NY: Springer Publishing Company, Inc.; 2010.

[41] Kucher K , Johns D , Maier D , et al. First‐in‐man intrathecal application of neurite growth‐promoting anti‐nogo‐a antibodies in acute spinal cord injury. Neurorehab Neur Repair. 2018;32:578‐589. 10.1177/1545968318776371 29869587

