# Supplementary material for: Combined with anti‐Nogo‐A antibody treatment, BDNF did not compensate the extra deleterious motor effect caused by large size cervical cord hemisection in adult macaques
Source: CNS Neurosci Ther. 2019 Aug 16;26(2):260–9. doi: 10.1111/cns.13213 (PMC6978268; doi:10.1111/cns.13213)
Supplement: Supplementary file 1 [file CNS-26-260-s001.docx]

**Supporting material**

**Further rationale for combining BDNF with the anti-Nogo-A antibody treatment**

Although the literature contains a large diversity of results and complexities as far as the effects of BDNF on neuroregeneration are concerned (see Supplementary Discussion below), there was still strong support toward a promising outcome, which prompted the present study associating BDNF with the anti-Nogo-A antibody. Among others, the study of Bareyre et al. (2002) represented a strong incentive, considering that the BDNF gene was found to be the most upregulated gene in rats subjected to unilateral CS injury and treated with anti-Nogo-A antibodies. In addition, we speculated that BDNF may have a broader spectrum of actions in the motor cortex than NT-3 for instance, acting also on e.g. cortico-bulbar or callosal projections, or even on interneurons, all of which could, by enhanced plasticity, contribute to the restoration of the complex hand function. Moreover, BDNF was used in preclinical trials of stroke. Finally, at the time of the study, BDNF was more accessible for us than NT-3.

**Supplementary Methods**

*Surgical procedures*

As previously reported in detail (Wannier et al., 2005; Schmidlin et al., 2004, 2005; Freund et al., 2006, 2007, 2009; Beaud et al., 2012), the anaesthesia was induced by intramuscular (i.m.) injection of ketamine (Ketalar®; Parke-Davis, 5 mg/kg, i.m.) and atropine was injected to reduce bronchial secretions (0.05 mg/kg, i.m.). Before surgery, the animal was treated with the analgesic Carprofen (Rymadil®, 4 mg/kg, s.c.). Then, a continuous perfusion of a mixture of propofol 1% (Fresenius®) and a 4% glucose solution (1 volume of Propofol and 2 volumes of glucose solution) was delivered through an intravenous catheter placed in the femoral vein in order to maintain a deep anaesthesia. Usually, the level of anaesthesia remained deep and stable at a rate of venous perfusion of Propofol/glucose mixture of 0.1 ml/min/kg. When the animal was deeply anaesthetized, it was placed in a stereotaxic headholder, using ear bars covered at their tip with local anaesthetic. Surgery was carried out under aseptic conditions. During the surgery, different parameters were monitored: heart rate, respiration rate, expired CO_2_, arterial O_2_ saturation and body temperature. An extra intravenous bolus of 0.5 mg of ketamine diluted in saline (0.9%) was added at potentially more painful steps of the surgical procedure, such as laminectomy. In later experiments, ketamine was added to the perfusion solution and delivered throughout surgery (0.0625 mg/min/kg).

Placed in a ventral decubitus position, the animal had a pillow under the chest, and its head was kept in a flexed position ventrally to expose the spinal processes from C2 to Th1. After a vertical midline skin incision, the paravertebral muscles were retracted and the laminae of segments C6, C7 and Th1 were dissected. A complete C6 laminectomy and an upper C7 hemi-laminectomy were then performed. The ligamentum flavum was removed in order to expose the dura mater, which was incised longitudinally. Observations on available anatomical material indicated that the transition level between the cervical spinal segments 7 and 8 was covered by the 6^th^ cervical vertebra. The dorsal root entry zone at the C7/C8 border was then identified, providing an anatomical landmark for placing a surgical blade (No. 11; Paragon®), which was used to perform an incomplete hemi-section of the cervical cord which aimed to completely cut the dorsolateral funiculus. The surgical blade was inserted 4 mm in depth perpendicularly to the main axis of the spinal cord, and the section was prolonged laterally to cut the dorsolateral funiculus. In most cases, such a section completely interrupted the CS tract unilaterally. The lesion is located at C7 level, caudal with respect to the main pool of biceps motoneurons but rostral to the pools of triceps, forearm, and hand muscle motoneurons (Jenny and Inukai 1983). The muscles and the skin were sutured and the animal recovered from anaesthesia usually 15-30 min after cessation of the venous perfusion with propofol. The animal was treated post-operatively with an antibiotic (Ampicilin 10%, 30mg/kg, s.c.). During the week following the surgery, Carprofen (4 my/kg) was given daily. After the spinal lesion, the animal was kept alone in a separate cage for a few days in order to ensure a careful survey of its health status. Two to five days after the surgery, the animal was replaced in the housing room with the other monkeys.

*Treatments and groups of monkeys*

The control antibody was a purified IgG of a mouse mAb directed against wheat auxin (AMS Biotechnology, Oxon, United Kingdom). (14.8 mg in 4 weeks), administered to 6 monkeys (Mk-CC, Mk-CP, Mk-CG, Mk-CS, Mk-CB, Mk-CH) derived from previous studies (Freund et al., 2006, 2007, 2009). Wheat auxin is an antigen that is not present in mammalian organs including the CNS. The antibody did not show any cross reactivity with or binding to mammalian CNS tissues. Seven monkeys (Mk-AS, MK-AF, Mk-AP, Mk-AA, Mk-AM, Mk-AK and Mk-AC), taken for comparison from previous studies (Freund et al., 2006, 2007, 2009) were implanted with a single pump delivering the anti-Nogo-A antibody only during 4 weeks (Freund et al., 2009: their Table 1). The characterization of the anti-Nogo-A antibodies was reported previously (see Oertle et al., 2003; Weinmann et al., 2006; Freund et al., 2007). In the anti-Nogo-A antibody alone group, two antibodies were used, 11C7 and hNogoA (Freund et al., 2006, 2007, 2009). 11C7 is a mouse monoclonal antibody, generated against rat Nogo-A, and is an IgG1. hNogoA is a humanized antibody, originally mouse, where large parts are replaced by human sequences (see Freund et al., 2009; see also Kucher et al., 2018).

Five animals newly introduced in the present report on the behavioural point of view (Mk-ABB, Mk-ABMx, Mk-ABMa, Mk-ABP and Mk-ABS) were implanted with two pumps, one delivering BDNF (1.4mg in 4 weeks) and the second the anti-Nogo-A antibody (14.8 mg in 4 weeks). The pumps were removed after 4 weeks, and we checked that they were empty. The biological activity of the infused BDNF was not tested (in the CSF), but BDNF is known to bind rapidly to CNS tissue. Its pharmacokinetic in the CNS is not known and must be very complex due to the presence of different receptor types as well as high unspecific sticking. The protocol accounted for this by using a continuous infusion during the entire treatment period. Testing CSF at specific time points would be required to obtain precise information, a procedure which is not optimal in monkeys, who’s behavioral performance was tested daily after the offset of the treatment.

Two control monkeys (Mk-CBo and Mk-CGa, performed at a later stage than those involved in Freund et al. (2006, 2007 and 2009), subjected to cervical hemi-section and which received the control antibody, were excluded from the present behavioural analysis. The cervical lesion was located too caudal (T1 or below) and therefore the deficits were modest: the two animals successfully grasped with the affected hand a few pellets already 2 days post-lesion (no score drop to zero) and fully recovered (100%) their manual dexterity in about 10 days. This is in sharp contrast to all other previous control antibody monkeys (n=6), which exhibited a score of zero for a few days post-lesion, followed by an incomplete (>100%) functional recovery reached only after 28-76 days (post-lesion plateau). Moreover, two monkeys (Mk-AG and Mk-AT) subjected to cervical cord lesion and to anti-Nogo-A antibody alone treatment were not considered either, as the lesion was also too caudal. These four discarded monkeys were however behaviourally analysed at the same time as the five monkeys with the combined treatment, which allowed blinding the daily experimenter with respect to the presence/absence/type of treatment (Table 1). The four discarded monkeys were however kept for neuroanatomical tracing analyses, data to be presented elsewhere.

*Histology*

After removal of the pumps, the animals continued to be behaviourally tested until they reached a stable post-lesion behavioural score. At that point, approximately three months before sacrifice of the animal, the anterograde tracer Biotinylated Dextran Amine (BDA; Molecular Probe®, Eugene, OR, USA) was injected in the primary motor cortex hand area on the contralesional hemisphere using Hamilton syringes in order to stain the CS tract as previously described (Freund et al., 2007; Beaud et al., 2008).

At the end of the survival period, the animals were sacrificed under deep (lethal) anaesthesia (90 mg sodium pentobarbital/kg body weight, i.p.) by transcardiac perfusion with 0.9% saline (400 ml). The perfusion was continued with fixative (3 litres of 4% phosphate-buffered paraformaldehyde in 0.1M phosphate buffer, pH 7.6) and solutions (2 litres each) of the same fixative containing increasing concentrations of sucrose (10%, 20% and 30%). The brain and the spinal cord were dissected and placed in a 30% solution of sucrose (in phosphate buffer) for cryo-protection for 7 days. Frozen sections (50 µm thick) of the brain were cut in the frontal plane, whereas frozen sections (50 µm thick) of cervical cord (approximately segments C6-T3) were cut in the parasagittal longitudinal plane and collected in three series for later histological processing. Upper cervical segments and lower thoracic spinal segments were cut in the frontal plane at 50 µm thickness, and sections were also collected in three series. BDA staining was revealed in one series of the spinal cord sections. The second series of spinal cord sections was immunohistochemically processed to visualize corticospinal axons in the spinal cord using the marker SMI-32 (Covance, Berkeley, CA, USA; see also Campbell and Morrison, 1989). These series of spinal cord sections were used to reconstruct the location and the extent of the cervical cord lesion as described in detail in another report (Wannier et al., 2005) (Fig. 1C). In addition to this parameter and as already introduced in Freund et al. (2009), the volume of each lesion was measured into the scar of SMI-32-stained sections in order to consider the extent of the lesion in 3-D rostro-caudal axis (Table 1 and Fig. 2) (Freund et al., 2009). The SMI-32 staining was chosen to reconstruct the extent of the lesion because the lesion contour was better defined, as compared to BDA staining. For all the anatomical analyses, a light microscope (Olympus) and Neurolucida software (MicroBrightField, Inc., Colchester, VT, USA) were used.

**Supplementary discussion**

A limitation of the present study, though common for non-human primates protocols for ethical reasons, is the low number of monkeys, especially in the group of combined treatment (n=5 versus 6-7 in the other 2 groups). Nevertheless, the data derived from these 5 monkeys are largely consistent among them, exhibiting differences in term of functional recovery with the other 2 groups of monkeys (Table 2). Another limitation, for the same reason as above, is the absence of a group of monkeys treated with BDNF alone. At that step, the effect of BDNF on the present model of SCI remains unknown. Finally, in the combined treatment group, the two treatments were delivered simultaneously leaving open the possibility of a better outcome if they were optimally adjusted in time, although the best schedule is unknown.

Nogo-A is a major neurite growth inhibiting molecule (Fawcett 2002; Oertle et al., 2003; Gonzenbach et al., 2010; Schwab 2010). Following a spinal cord injury in rats or primates, treating the lesioned animals with antibodies directed against Nogo-A promotes sprouting and regeneration of CS fibers and improves functional recovery (Bregman et al., 1995; Brösamle et al., 2000; Fouad et al., 2004; Freund et al., 2006, 2007, 2009; Liebscher et al., 2005; Schnell and Schwab 1990; Thallmair et al., 1998). In previous investigations in adult macaques (Freund et al., 2006, 2009), we have reported that treating SCI monkeys with a control antibody leads to a lower level of functional recovery than that observed when treating SCI animals with an antibody neutralizing Nogo-A.

BDNF, like the other neurotrophic factors, has been shown to contribute to neuronal survival, axonal growth, synaptic plasticity and neurotransmission. The receptor of BDNF, *trkB*, is expressed in normal and axotomized CS neurons of rodents and of primates (Giehl et al., 2001; Lu et al., 2001;Ohira et al., 1999; Okuno et al., 1999; Zhang et al., 2007) and grafting BDNF/NT-3-secreting cells within the sites of a C7 hemi-section in primates promotes regeneration and hinders the atrophy of the CS soma cells (Brock et al., 2010). In addition, rats subjected to a high unilateral lesion of the CST and treated with an anti-Nogo-A antibody show compensatory sprouting within the spinal cord which is associated with an increase in gene expression of BDNF (Bareyre et al., 2002). These studies suggest that combining the neutralization of Nogo-A to applications of BDNF could stimulate axonal regeneration and/or sprouting of CS neurons and thereby provide a structural basis for improving functional recovery. Such synergy was not confirmed in the present study on macaques after SCI.

Application of BDNF to lesioned CST neurons in rats promotes collateral spouting of the axotomized CS axons rostrally to the lesion (Hiebert et al., 2002; Vavrek et al., 2006). Likewise, overexpression of BDNF in the rat sensorimotor cortex together with overexpression of NT-3 at the lumbar spinal level significantly amplifies axonal sprouting for the injured CST compared to that induced by the NT-3 alone (Zhou and Shine 2003). Moreover, injections of BDNF-hypersecreting human mesenchymal stem cells rostrally and caudally to a thoracic lesion in adult rat result in an improved locomotor recovery and an increased sprouting of CS and serotonergic fibers, as well as to an increase of CS neurons cell survival in M1 cortex (Sasaki et al., 2009). In the same way, other studies have shown that the delivery of BDNF protect injured CS neurons and can improve functional recovery (Giehl and Tetzlaff 1996; Hammond et al., 1999; Jakeman et al., 1998; Kim and Jahng 2004; Namiki et al., 2000). However, as various studies also fail to observe regenerating effects of BDNF on CS neurons, the effects of BDNF on these neurons remain a controversy. For instance, in adult rats, transplants of fetal spinal cord and BDNF administrated two weeks after SCI do not significantly increase the amount of spared CS axons rostrally and caudally to the lesion whereas transplants combined with NT-3 do (Iarikov et al., 2007). Likewise, BDNF-secreting grafts elicited no growth or limited growth responses of CS nerve fibers in adult rats after SCI and fail to improve functional recovery of primary motor functions (Lu et al., 2001; Nakahara et al., 1996; Shumsky et al., 2003; Tobias et al., 2003).

Very little is known on the effects obtained by combining neurotrophic factors to antibodies neutralizing Nogo-A and the few investigations dealing with that topic have also led to ambiguous results. For instance, in neonatal rats, the regeneration of injured optic nerve fibers is improved by associating BDNF with an antibody neutralizing Nogo-A (Weibel et al., 1994, 1995). Similarly, the combined application of an antibody neutralizing Nogo-A with the ciliary neurotrophic factor (CTNF) enhances axonal regeneration of transected retinal ganglions axons (Cui et al., 2004). In contrast, whereas sprouting of CS axons was enhanced by combining an anti-Nogo-A antibody with injections of NT-3 in young adult rats subjected to spinal cord injury, no effects were observed with the combination of the same antibody with injections of BDNF (Schnell et al., 1994). As we failed to observe a synergistic effect when BDNF is added to the anti-Nogo-A antibody in macaques after SCI, our observations are thus in line with this last report in rats. As exogenous applications of neurotrophins, including BDNF, are known to act differentially on different categories of neurons such as raphespinal, coeruleospinal or CS axons (Bregman et al., 1997), it is not excluded that other motor attributes which do not rely heavily on CS projections, may improve as a result of the combined treatment.

**References for supporting material**

Bareyre,F.M., Haudenschild,B. & Schwab,M.E. (2002) Long-lasting sprouting and gene expression changes induced by the monoclonal antibody IN-1 in the adult spinal cord. *J.Neurosci.*, **22**, 7097-7110.

Beaud,M.L., Schmidlin,E., Wannier,T., Freund,P., Bloch,J., Mir,A., Schwab,M.E. & Rouiller,E.M. (2008) Anti-Nogo-A antibody treatment does not prevent cell body shrinkage in the motor cortex in adult monkeys subjected to unilateral cervical cord lesion. *BMC.Neurosci.*, **9**, 5.

Beaud M.L., Rouiller E.M., Bloch J., Mir A., Schwab M.E., Wannier T. & Schmidlin E.. (2012) [Invasion of lesion territory by regenerating fibers after spinal cord injury in adult macaque monkeys.](https://www.ncbi.nlm.nih.gov/pubmed/23036616) *Neuroscience*, 227, 271-282.

Bregman,B.S., Kunkel-Bagden,E., Schnell,L., Dai,H.N., Gao,D. & Schwab,M.E. (1995) Recovery from spinal cord injury mediated by antibodies to neurite growth inhibitors. *Nature*, **378**, 498-501.

Bregman,B.S., McAtee,M., Dai,H.N. & Kuhn,P.L. (1997) Neurotrophic factors increase axonal growth after spinal cord injury and transplantation in the adult rat. *Exp.Neurol.*, **148**, 475-494.

Brock,J.H., Rosenzweig,E.S., Blesch,A., Moseanko,R., Havton,L.A., Edgerton,V.R. & Tuszynski,M.H. (2010) Local and remote growth factor effects after primate spinal cord injury. *J.Neurosci.*, **30**, 9728-9737.

Brösamle,C., Huber,A.B., Fiedler,M., Skerra,A. & Schwab,M.E. (2000) Regeneration of lesioned corticospinal tract fibers in the adult rat induced by a recombinant, humanized IN-1 antibody fragment. *J.Neurosci.*, **20**, 8061-8068.

Campbell,M.J. & Morrison,J.H. (1989) Monoclonal antibody to neurofilament protein (SMI-32) labels a subpopulation of pyramidal neurons in the human and monkey neocortex. *J Comp Neurol*, **282**, 191-205.

Cui,Q., Cho,K.S., So,K.F. & Yip,H.K. (2004) Synergistic effect of Nogo-neutralizing antibody IN-1 and ciliary neurotrophic factor on axonal regeneration in adult rodent visual systems. *J.Neurotrauma*, **21**, 617-625.

Fawcett,J. (2002) Repair of spinal cord injuries: where are we, where are we going? *Spinal Cord.*, **40**, 615-623.

Fouad,K., Klusman,I. & Schwab,M.E. (2004) Regenerating corticospinal fibers in the Marmoset (*Callitrix jacchus*) after spinal cord lesion and treatment with the anti-Nogo-A antibody IN-1. *Eur.J.Neurosci.*, **20**, 2479-2482.

Freund,P., Schmidlin,E., Wannier,T., Bloch,J., Mir,A., Schwab,M.E. & Rouiller,E.M. (2006) Nogo-A-specific antibody treatment enhances sprouting and functional recovery after cervical lesion in adult primates. *Nature Med.*, **12**, 790-792.

Freund,P., Wannier,T., Schmidlin,E., Bloch,J., Mir,A., Schwab,M.E. & Rouiller,E.M. (2007) Anti-Nogo-A antibody treatment enhances sprouting of corticospinal axons rostral to a unilateral cervical spinal cord lesion in adult macaque monkey. *J.Comp Neurol.*, **502**, 644-659.

Freund,P., Schmidlin,E., Wannier,T., Bloch,J., Mir,A., Schwab,M.E. & Rouiller,E.M. (2009) Anti-Nogo-A antibody treatment promotes recovery of manual dexterity after unilateral cervical lesion in adult primates--re-examination and extension of behavioral data. *Eur.J.Neurosci.*, **29**, 983-996.

Giehl,K.M. & Tetzlaff,W. (1996) BDNF and NT-3, but not NGF, prevent axotomy-induced death of rat corticospinal neurons *in vivo*. *Eur.J.Neurosci.*, **8**, 1167-1175.

Giehl,K.M., Röhrig,S., Bonatz,H., Gutjahr,M., Leiner,B., Bartke,I., Yan,Q., Reichardt,L.F., Backus,C., Welcher,A.A., Dethleffsen,K., Mestres,P. & Meyer,M. (2001) Endogenous brain-derived neurotrophic factor and neurotrophin-3 antagonistically regulate survival of axotomized corticospinal neurons *in vivo*. *J.Neurosci.*, **21**, 3492-3502.

Gonzenbach,R.R., Gasser,P., Zorner,B., Hochreutener,E., Dietz,V. & Schwab,M.E. (2010) Nogo-A antibodies and training reduce muscle spasms in spinal cord-injured rats. *Ann.Neurol.*, **68**, 48-57.

Hammond,E.N.L., Tetzlaff,W., Mestres,P. & Giehl,K.M. (1999) BDNF, but not NT-3, promotes long-term survival of axotomized adult rat corticospinal neurons *in vivo*. *NeuroReport*, **10**, 2671-2675.

Hiebert,G.W., Khodarahmi,K., McGraw,J., Steeves,J.D. & Tetzlaff,W. (2002) Brain-derived neurotrophic factor applied to the motor cortex promotes sprouting of corticospinal fibers but not regeneration into a peripheral nerve transplant. *J.Neurosci.Res.*, **69**, 160-168.

Iarikov,D.E., Kim,B.G., Dai,H.N., McAtee,M., Kuhn,P.L. & Bregman,B.S. (2007) Delayed transplantation with exogenous neurotrophin administration enhances plasticity of corticofugal projections after spinal cord injury. *J.Neurotrauma*, **24**, 690-702.

Jakeman,L.B., Wei,P., Guan,Z. & Stokes,B.T. (1998) Brain-derived neurotrophic factor stimulates hindlimb stepping and sprouting of cholinergic fibers after spinal cord injury. *Exp.Neurol.*, **154**, 170-184.

Jenny,A.B. & Inukai,J. (1983) Principles of motor organization of the monkey cervical spinal cord. *J.Neurosci.*, **3**, 567-575.

Kim,D.H. & Jahng,T.A. (2004) Continuous brain-derived neurotrophic factor (BDNF) infusion after methylprednisolone treatment in severe spinal cord injury. *J.Korean Med Sci.*, **19**, 113-122.

Kucher K, Johns D, Maier D, Abel R, Badke A, Baron H, Thietje R, Casha S, Meindl R, Gomez-Mancilla B, Pfister C, Rupp R, Weidner N, Mir A, Schwab ME, Curt A. (2018) [First-in-Man Intrathecal Application of Neurite Growth-Promoting Anti-Nogo-A Antibodies in Acute Spinal Cord Injury.](https://www.ncbi.nlm.nih.gov/pubmed/29869587) *Neurorehab Neur Repair* **32**:578-589.

Liebscher,T., Schnell,L., Schnell,D., Scholl,J., Schneider,R., Gullo,M., Fouad,K., Mir,A., Rausch,M., Kindler,D., Hamers,F.P.T. & Schwab,M.E. (2005) Nogo-A antibody improves regeneration and locomotion of spinal cord-injured rats. *Ann.Neurol.*, **58**, 706-719.

Lu,P., Blesch,A. & Tuszynski,M.H. (2001) Neurotrophism without neurotropism: BDNF promotes survival but not growth of lesioned corticospinal neurons. *J.Comp.Neurol.*, **436**, 456-470.

Namiki,J., Kojima,A. & Tator,C.H. (2000) Effect of brain-derived neurotrophic factor, nerve growth factor, and neurotrophin-3 on functional recovery and regeneration after spinal cord injury in adult rats. *Journal of Neurotrauma*, **17**, 1219-1230.

Nakahara,Y., Gage,F.H. & Tuszynski,M.H. (1996) Grafts of fibroblasts genetically modified to secrete NGF, BDNF, NT-3, or basic FGF elicit differential responses in the adult spinal cord. *Cell Transplant.*, **5**, 191-204.

Oertle,T., Van der Haar,M.E., Bandtlow,C.E., Robeva,A., Burfeind,P., Buss,A., Huber,A.B., Simonen,M., Schnell,L., Brosamle,C., Kaupmann,K., Vallon,R. & Schwab,M.E. (2003) Nogo-A inhibits neurite outgrowth and cell spreading with three discrete regions. *J.Neurosci.*, **23**, 5393-5406.

Ohira,K., Shimizu,K. & Hayashi,M. (1999) Change of expression of full-length and truncated TrkBs in the developing monkey central nervous system. *Brain Res.Dev.Brain Res.*, **112**, 21-29.

Okuno,H., Tokuyama,W., Li,Y.X., Hashimoto,T. & Miyashita,Y. (1999) Quantitative evaluation of neurotrophin and trk mRNA expression in visual and limbic areas along the occipito-temporo-hippocampal pathway in adult macaque monkeys. *J.Comp Neurol.*, **408**, 378-398.

Sasaki,M., Radtke,C., Tan,A.M., Zhao,P., Hamada,H., Houkin,K., Honmou,O. & Kocsis,J.D. (2009) BDNF-hypersecreting human mesenchymal stem cells promote functional recovery, axonal sprouting, and protection of corticospinal neurons after spinal cord injury. *J.Neurosci.*, **29**, 14932-14941.

Schmidlin,E., Wannier,T., Bloch,J. & Rouiller,E.M. (2004) Progressive plastic changes in the hand representation of the primary motor cortex parallel incomplete recovery from a unilateral section of the corticospinal tract at cervical level in monkeys. *Brain Research*, **1017**, 172-183.

Schmidlin,E., Wannier,T., Bloch,J., Belhaj-Saïf,A., Wyss,A. & Rouiller,E.M. (2005) Reduction of the hand representation in the ipsilateral primary motor cortex following unilateral section of the corticospinal tract at cervical level in monkeys. *BMC Neuroscience*, **6:56.**

Schnell,L. & Schwab,M.E. (1990) Axonal regeneration in the rat spinal cord produced by an antibody against myelin-associated neurite growth inhibitors. *Nature*, **343**, 269-272.

Schnell,L., Schneider,R., Kolbeck,R., Barde,Y.A. & Schwab,M.E. (1994) Neurotrophin-3 enhances sprouting of corticospinal tract during development and after adult spinal cord lesion. *Nature*, **367**, 170-173.

Schwab,M.E. (2010) Functions of Nogo proteins and their receptors in the nervous system. *Nat.Rev.Neurosci.*, **11**, 799-811.

Shumsky,J.S., Tobias,C.A., Tumolo,M., Long,W.D., Giszter,S.F. & Murray,M. (2003) Delayed transplantation of fibroblasts genetically modified to secrete BDNF and NT-3 into a spinal cord injury site is associated with limited recovery of function. *Experimental Neurology*, **184**, 114-130.

Thallmair,M., Metz,G.A.S., Z'Graggen,W.J., Raineteau,O., Kartje,G.L. & Schwab,M.E. (1998) Neurite growth inhibitors restrict plasticity and functional recovery following corticospinal tract lesions. *Nature Neurosci.*, **1**, 124-131.

Tobias,C.A., Shumsky,J.S., Shibata,M., Tuszynski,M.H., Fischer,I., Tessler,A. & Murray,M. (2003) Delayed grafting of BDNF and NT-3 producing fibroblasts into the injured spinal cord stimulates sprouting, partially rescues axotornized red nucleus neurons from loss and atrophy, and provides limited regeneration. *Experimental Neurology*, **184**, 97-113.

Vavrek,R., Girgis,J., Tetzlaff,W., Hiebert,G.W. & Fouad,K. (2006) BDNF promotes connections of corticospinal neurons onto spared descending interneurons in spinal cord injured rats. *Brain*, **129**, 1534-1545.

Wannier,T., Schmidlin,E., Bloch,J. & Rouiller,E.M. (2005) A unilateral section of the corticospinal tract at cervical level in primate does not lead to measurable cell loss in motor cortex. *Journal of Neurotrauma*, **22**, 703-717.

Weibel,D., Cadelli,D. & Schwab,M.E. (1994) Regeneration of lesioned rat optic nerve fibers is improved after neutralization of myelin-associated neurite growth inhibitors. *Brain Res.*, **642**, 259-266.

Weibel,D., Kreutzberg,G.W. & Schwab,M.E. (1995) Brain-derived neurotrophic factor (BDNF) prevents lesion-induced axonal die-back in young rat optic nerve. *Brain Res.*, **679**, 249-254.

Weinmann,O., Schnell,L., Ghosh,A., Montani,L., Wiessner,C., Wannier,T., Rouiller,E., Mir,A. & Schwab,M.E. (2006) Intrathecally infused antibodies against Nogo-A penetrate the CNS and downregulate the endogenous neurite growth inhibitor Nogo-A. *Mol.Cell Neurosci.*, **32**, 161-173.

Zhang,H.T., Li,L.Y., Zou,X.L., Song,X.B., Hu,Y.L., Feng,Z.T. & Wang,T.T. (2007) Immunohistochemical distribution of NGF, BDNF, NT-3, and NT-4 in adult rhesus monkey brains. *J.Histochem.Cytochem.*, **55**, 1-19.

Zhou,L. & Shine,H.D. (2003) Neurotrophic factors expressed in both cortex and spinal cord induce axonal plasticity after spinal cord injury. *J.Neurosci.Res.*, **74**, 221-226.
